# Stacked 3D RRAM Array with Graphene/CNT as Edge Electrodes

**DOI:** 10.1038/srep13785

**Published:** 2015-09-08

**Authors:** Yue Bai, Huaqiang Wu, Kun Wang, Riga Wu, Lin Song, Tianyi Li, Jiangtao Wang, Zhiping Yu, He Qian

**Affiliations:** 1 Institute of Microelectronics, Tsinghua University, Beijing, China, 100084; 2 Tsinghua National Laboratory for Information Science and Technology (TNList), Beijing, China, 100084; 3 Inner Mongolia University, Hohhot, Inner Mongolia, China, 010021; 4 State Key Laboratory of Low-Dimensional Quantum Physics, Department of Physics and Tsinghua-Foxconn Nanotechnology Research Center, Tsinghua University, Beijing 100084, China

## Abstract

There are two critical challenges which determine the array density of 3D RRAM: 1) the scaling limit in both horizontal and vertical directions; 2) the integration of selector devices in 3D structure. In this work, we present a novel 3D RRAM structure using low-dimensional materials, including 2D graphene and 1D carbon nanotube (CNT), as the edge electrodes. A two-layer 3D RRAM with monolayer graphene as edge electrode is demonstrated. The electrical results reveal that the RRAM devices could switch normally with this very thin edge electrode at nanometer scale. Meanwhile, benefited from the asymmetric carrier transport induced by Schottky barrier at metal/CNT and oxide/CNT interfaces, a selector built-in 3D RRAM structure using CNT as edge electrode is successfully fabricated and characterized. Furthermore, the discussion of high array density potential is presented.

The growing demands in high-density memories drive the rapid development of advanced memory technologies. As one of the most promising emerging non-volatile memory (NVM) devices, oxide-based resistive switching memory (RRAM) has attracted significant interests due to the super endurance, fast switching speed, low power consumption and good CMOS compatibility^1^,^2^,^3^,^4^,^5^,^6^,^7^. On the other hand, current flash technology also found a way to overcome its scaling limit by adopting three dimensional (3D) structure to achieve high density^8^,^9^. The 3D RRAM approach, which combines the advantages of excellent electrical performances in RRAM cell and high density of 3D configuration, becomes a very attractive candidate for next generation high density NVM applications^10^,^11^,^12^. Since various 3D RRAM structures have been proposed, in this article, the 3D RRAM structure specifically refers to a typical architecture which is shown in Fig. 1: the RRAM cell is consisted with vertical resistive switching layer on the side wall of the drilled hole, vertical metal pillar as one electrode, and the edge of metal plane as the other electrode. Figure 1 shows that, different from planar RRAM, the effective area, S_effective_, of 3D RRAM cell could be calculated as:


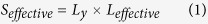


Where L_y_ and L_effective_ represent the thicknesses of the metal plane and the effective perimeter which is in touch with resistive switching layer at horizontal direction. For traditional metal plane electrode based 3D RRAM, since it has cylindrical shape with surrounding electrode, the L_effective_ could be calculated as:


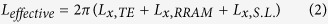


Where L_x,pillar_, L_x,RRAM_, L_x,S.L._ represent thicknesses of pillar electrode, resistive switching layer and selector layer respectively. Previous studies on planar RRAM structure have already shown that 3 nm RRAM device could deliver normal resistive switching performances^13^. Therefore, to evaluate the potential of 3D RRAM in competing with 3D NAND, two critical questions should be addressed. 1) Literatures have shown that metal plane edge electrode provides significant scaling benefit in vertical direction^14^,^15^. What is the scaling limit of L_y_? 2) How to find an efficient method to integrate a selector device? Similar to 2D crossbar structure, 3D RRAM array also has sneak path current issue, as shown in Fig. 1, which could be addressed by integrating a selector^16^. A few research groups chose an oxide layer insertion to create a selector in series^10^,^17^. However, the selector layer thicknesses (L_x,S.L._) in those approaches are even larger than that of switching layer (L_x,RRAM_), which limits the size scaling in horizontal direction^10^.

Recently, carbon based low dimensional materials, including carbon nanotube(CNT) and graphene, have drawn significant scientific and technological interests as the emerging interconnect solution^18^,^19^. Being only one or a few atomic layer thick, they represent the ultimate limit of size^20^. Meanwhile, the previous studies have shown that CNT and graphene electrode could bring better performance in planar RRAM structure^21^,^22^. In this work, we used graphene and CNT as the electrode materials to investigate the scaling limit in 3D RRAM structure. This study could answer if 3D RRAM cell would operate at sub-nanometer scale in vertical direction. Meanwhile, benefited from Schottky barrier, a selector could be self-integrated at the interface between resistive switching layer and CNT electrode. Through this innovative structure, the selector layer could be avoided and higher density could be achieved with smaller pitch in horizontal direction.

## Results and Disscussion

### Scaling in vertical direction (L_y_ scaling down)

Two-layer 3D Ta_2_O_5-x_/TaO_y_ RRAM cells are fabricated using monolayer graphene as the edge electrode. Figure 2(a) shows the schematic view of the device structure. The monolayer graphene was grown on the Pt substrate using CVD method and transferred to SiO_2_ substrate by an electrochemical approach^23^. The Pt pillar and graphene layer serve as pillar electrode and edge electrode respectively, while the transitional metal oxide (TMO) resistive switching layer is located vertically on the sidewall between pillar electrode and edge electrode. Pd is chosen as the contact metal to graphene for signal output. The cross-sectional TEM image in Fig. 2(b) and magnified false-color EELS map in Fig. 2(c) show the typical structure of 3D Ta_2_O_5-x_/TaO_y_ RRAM devices using monolayer graphene as the edge electrode. Figure 2(d) shows the optical top view of the monolayer graphene with pillar electrode and metal contact. Raman spectrum analysis was applied on the grown graphene layer, as shown in Fig. 2(e). The position and shape of G and 2D peaks in the spectrum confirm that the graphene used in the 3D RRAM structure is a monolayer graphene with thickness of ~0.3 nm.

The electrical performance of two-layer 3D Ta_2_O_5-x_/TaO_y_ RRAM with graphene edge electrode is shown in Fig. 3. The successful forming operations of both top and bottom layer cells are shown in Fig. 3(a), with a forming voltage of −6 V. Figure 3(b) shows the typical double I-V DC sweeping curves of top and bottom layer cells, with the SET and RESET voltages at −4.5 V and 4.5 V respectively. It shows a self-compliance property without external current limiting device in forming and SET operations. Comparing to pervious 3D RRAM with Pt metal plane as the edge electrode^11^, graphene edge electrode device has much smaller operation current (μA with graphene edge electrode vs. ~mA with Pt metal plan edge electrode). One possible explanation is that, when the TaO_y_ oxide layer contacts with graphene layer during deposition process, the edge of graphene could be oxidized. The previous studies have shown that, in the graphene/Ta_2_O_5-x_/TaO_y_/graphene system, a certain concentration of epoxide groups were grafted onto the basal plane of graphene^24^. As a result, the graphene is partially oxidized which increases contact resistance significantly. As a result, a high resistance region is generated at the interface between TaO_y_ and graphene. Serving as an internal resistor, this high resistance region helps control the overshoot current and achieves self-compliance property during SET and forming operations. Figure 3(c) shows the retention test results where both HRS at 100 MΩ and LRS at 10 MΩ could be kept stable for more than 10^4^ s at 85 °C.

Furthermore, It is necessary to investigate the mechanism when the edge electrode scaling to sub-nanometer. Therefore, The temperature dependent transport characteristics in HRS and LRS are studied to understand the conduction mechanism. Figure 4 shows that electrical measurement results in HRS are well fitted with Schottky barrier emission. While the electron transport is facilitated by electron hopping for LRS state. The conduction mechanism is quite similar with our previous study^11^. Therefore we propose a possible switching mechanism of graphene electrode based 3D RRAM: The mechanism is still caused by the formation/rupture of conductive filaments. And the filaments formation/rupture happen at the pillar electrode Pt/Ta_2_O_5-x_ interface, other than graphene/TaO_y_ interface. During forming process, the oxygen vacancies in TaO_y_ layer move to Ta_2_O_5-x_ layer, at the Pt/Ta_2_O_5-x_ the conductive filaments are formed. On the graphene/TaO_y_ side, there are a lot of oxygen vacancies in TaO_y_ which has good conductivity. Electrons could transport from graphene to TaO_y_ easily and enough current could be supplied. Since the graphene electrode has little effects on this process, it is possible that the filament size is still in several-nanometers order, which could be larger than graphene electrode.

The above physical characterizations and electrical results prove the proposed 3D RRAM cell could switch successfully with monolayer graphene as edge electrode. This ultra-small feature size (~0.3 nm) confirms the 3D RRAM could scale down to sub-nanometer in vertical direction.

### Scaling in horizontal direction (L_x,S.L._ scaling down)

**Figure 1:**
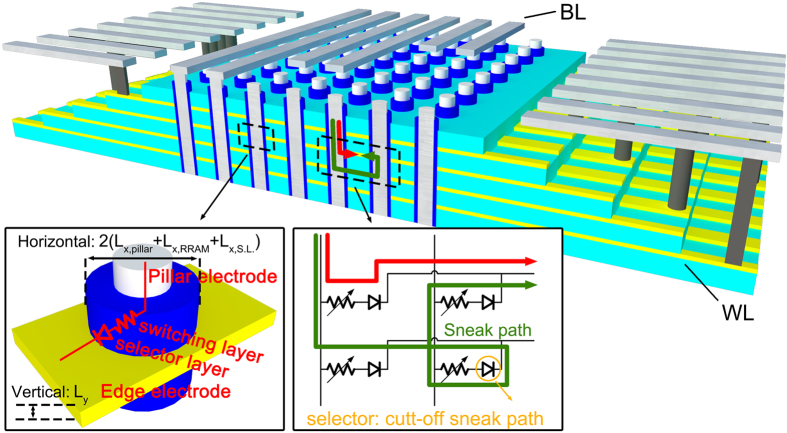
The typical schematic of 3D RRAM architecture. The sneak path currents exist in each vertical plane which dominates the maximum number of cells in the array. Therefore, the selector is a critical element to cut off the sneak path and achieve high density integration. The feature size scaling down of 3D RRAM can be divided into two parts: 1) the vertical direction decided by the thickness of metal plane; 2) the horizontal direction determined by the thickness of metal pillar, resistive switching layer and selector layer.

3D RRAM has more challenges of scaling down in horizontal direction. Due to the sneak path current issue, as shown in Fig. 1, RRAM array would not work without a selector device in series. In previous studies, NbO_2_^17^, TiO_x_^10^,^25^ have been tried as the selector layer material in 3D RRAM cells. However, in published experimental results, the thickness of selector layer is almost double of the resistive switching layer. This causes it very challenge to achieve the continuing hole dimension scaling. Therefore, creative solutions for selector devices integration in 3D RRAM structure are very much desired.

**Figure 2:**
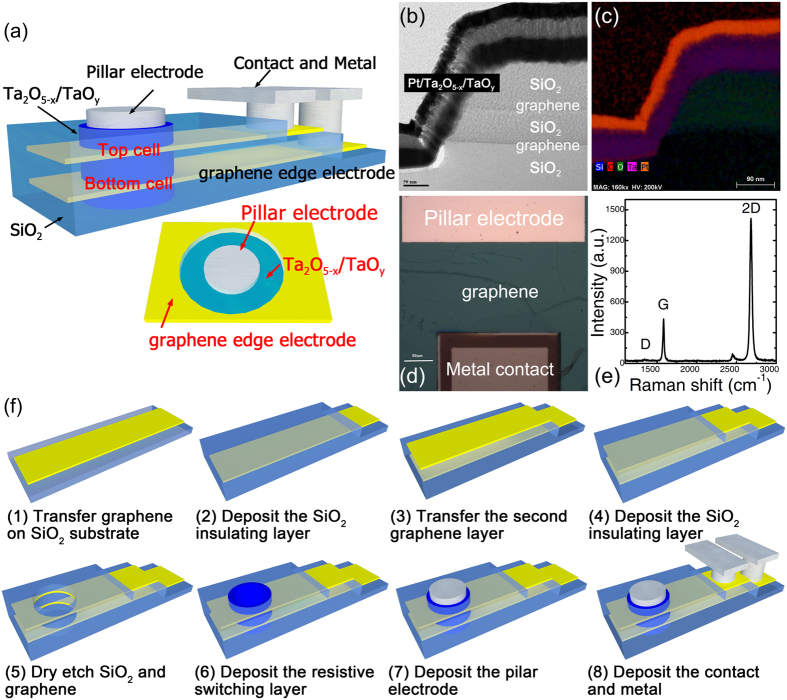
(**a**) The Schematic diagram of two-layer 3D Ta_2_O_5-x_/TaO_y_ RRAM with graphene edge electrode; the physical structure was characterized by (**b**) TEM image and (**c**) magnified false-color EELS map in cross-sectional view and (**d**) optical microscope image in top view; (**e**) the Raman spectra indicated the single layer graphene; (**f**) The fabrication flow of the single 3D RRAM cell.

**Figure 3:**
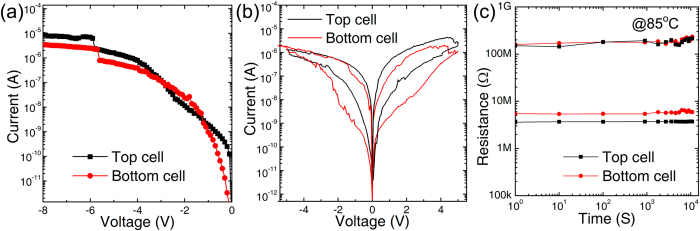
Electrical performance of two-layer 3D Ta_2_O_5-x_/TaO_y_ RRAM with graphene edge electrode: (**a**) Forming process of the cells in top and bottom layer which shows a self -compliance property without external device to limit the currents; (**b**) typical bipolar resistive switching; (**c**) retention measurement at 85 °C with 1 V read voltage.

**Figure 4:**
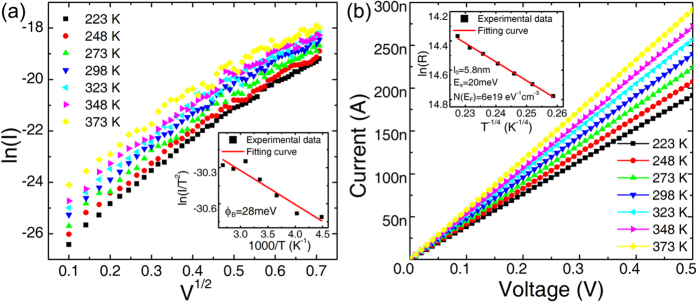
The conductive mechanism of 3D Ta_2_O_5-x_/TaO_y_ RRAM with graphene edge electrode in (a) HRS and (b) LRS, fitted to Schottky emission model and electron hopping model respectively. Insets show the fitting curves and corresponding fitting parameters.

**Figure 5:**
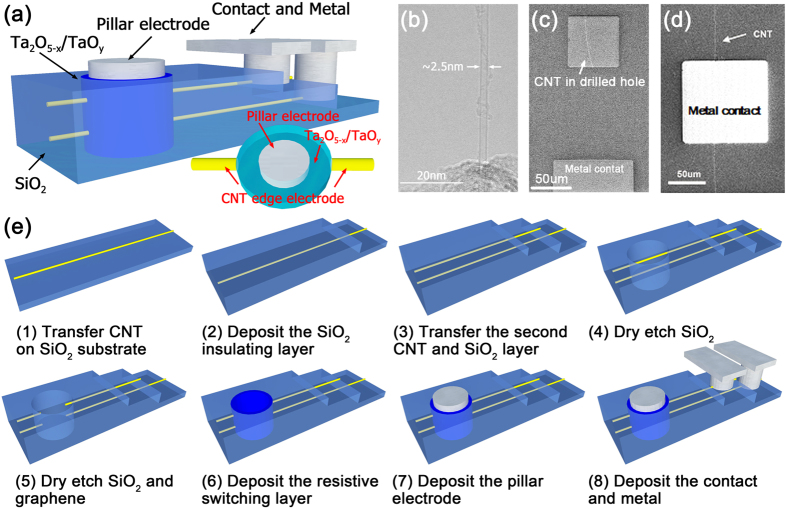
(**a**) Schematic diagram of 3D Ta_2_O_5-x_/TaO_y_ RRAM with CNT edge electrode; (**b**) TEM image of CNT with a diameter about 2.5nm; (**c**) The top view SEM image of the drilled hole after etching SiO_2_ layer without damaging the CNT electrode and (**d**) the metal contact Sc deposited on CNT; (**e**) The fabrication flow of the single 3D RRAM cell.

A novel approach of using CNT as the edge electrode and a self-integrated selector is proposed and devices are fabricated. Figure 5(a) shows the schematic view of the 3D Ta_2_O_5-x_/TaO_y_ RRAM using CNT as the edge electrode. The effective area S_effective_ in this case is further reduced, which closes to the magnitude of CNT cross-section area. The fabrication process is similar to that of 3D RRAM with graphene edge electrode, as shown in Fig. 5(e). The CNT was synthesized on SiO_2_ substrate directly by CVD method^26^. Figure 5(b) shows the TEM image of semiconducting CNT which confirms the single wall property with a diameter of 2.5 nm. Since CNT is tiny, it is very challenge to get good electrical contact between TMO and CNT. CNT could be etched away during the hole etching process and leaves no electrical contact or bad electrical contact between TMO and CNT. Two-step etching process was specially designed to deliver good electrical contact between TMO and CNT: 1) etching the SiO_2_ insulator layer using HF chemistry which wouldn’t damage the CNT, as shown in Fig. 5(c); 2) etching the CNT using low power oxygen plasma in the drilled hole. Our experimental results showed that this two-step etching process could deliver repeatable and controllable holes without damaging CNT. In this 3D RRAM structure, metal Sc was chosen as the contact metal to CNT for signal output, as shown in Fig. 5(d). It is worth to point out that the architecture of CNT edge electrode based 3D RRAM array is different from the traditional 3D RRAM array (as shown in Fig. 1). To access each cell, the architecture in Figure S1 of supplemental material should be adopted which is suitable for other nanowire materials.

**Figure 6:**
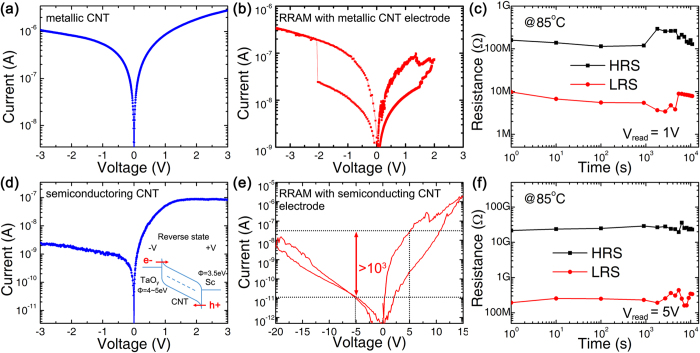
Electrical performance of 3D Ta_2_O_5-x_/TaO_y_ RRAM with metallic CNT edge electrode: (**a**) the Semi-log I-V curves of CNT between TaO_y_ and Sc electrode; (**b**) the typical bipolar resistive switching; (**c**) the retention measurement at 85 °C with 1 V read voltage. And electrical performance of device with semiconducting CNT BE: (**a**) the Semi-log I-V curves of CNT between TaO_y_ and Sc electrode, with the band-gap structure shown in the inset image; (**b**) the typical bipolar resistive switching with a rectification ratio more than 10^3^; (**c**) the retention measurement at 85 °C with 5 V read voltage.

Figure 6 shows the electrical performance of the 3D Ta_2_O_5-x_/TaO_y_ RRAM with CNT edge electrode. Both metallic and semiconducting CNTs were investigated. The metallic CNT with TaO_y_ and Sc electrodes shows a near-ohmic behaviour as shown in Fig. 6(a). Following the transport property of CNT, the fabricated 3D RRAM device using metallic CNT as edge electrode has symmetrical I-V curve, as shown in Fig. 6(b). It confirms that 3D RRAM with metallic CNT edge electrode could switch successfully similar to the cell with graphene edge electrode. The cell retention measurement result shows that both HRS and LRS could be kept stable at 100 MΩ and 10 MΩ for more than 10^4^ s at 85 °C, as shown in Fig. 6(c).

For semiconducting CNT as edge electrode case, a totally different transport property was observed, as shown in Fig. 6(d). It is believed that this asymmetrical I-V curve originates from the Schottky barrier formed between metal and semiconducting CNT^27^. The Fermi level of Sc aligns, in an almost barrier-free manner, with the conductance band of CNT. While the valence band of CNT aligns with the Fermi level of TaO_y_. At reverse cut-off state with positive voltage on edge electrode and negative voltage on pillar electrode, the Schottky barriers block the hole transport at Sc/semiconducting CNT end and the electron transport at TaO_y_/semiconducting CNT end, as shown in the inset of Fig. 6(d). This built-in current rectifying characteristics of contacts between CNT with Sc and TaO_y_ could serve as the built-in bi-directional selector for 3D RRAM without inserting any additional selector layer.

By choosing the metal contact Sc and TMO deposition sequence, the built-in selector could reduce the over-shoot current and sneak-path current simultaneously: During SET process with negative voltage on pillar electrode, the build-in selector is under reverse bias, which reduces the over-shoot current and achieve better self-compliance property. While in RESET process, the selector under forward bias supplies enough driving current to assist the resistive switching process. Figure 6(e) shows the switching behaviour of 3D RRAM integrated with semiconducting CNT edge electrode. The asymmetrical I-V curve is totally different from that of the devices with graphene or metallic CNT edge electrode. The LRS resistance on positive read bias is 1000 times of that on negative read bias. This transport property could reduce the sneak path current efficiently. This is because that at least one of the cells on sneak path would have negative bias. For the asymmetrical I-V devices, the equivalent resistance on sneak path will be more than 1000 time of that on selected cell. Additionally, it is noticed that the switching voltage in device with semiconducting CNT edge electrode is about 10 times larger than that in device with metallic CNT edge electrode. A possible reason is that a large series resistance is introduced at the contact between semiconducting CNT and TMO layer, which causes the effective voltage across the RRAM reduced. In addition, the HRS and LRS of 3D RRAM with semiconducting CNT edge electrode could also be kept stable for more than 10^4^ s at 85 °C at 10 GΩ and 100 MΩ with 5 V read voltage, as shown in Fig. 6(f).

### Performance potential of 3D vertical RRAM array

Since the 3D RRAM has been experimentally demonstrated at single cell level, it is important to evaluate the performance potential at the array level. Next, a simulation study is conducted to evaluate the 3D RRAM architecture with metal plane/2D/1D nano-materials. A resistor network model is constructed using SPICE method assisted by MATLAB. Three types of 3D RRAM cells, using Pt edge electrode, graphene edge electrode and semiconducting CNT edge electrode, have been compared based on measured data. Moreover, the interconnect resistance between neighboring cells is taken into account, following previous literature^28^. The simulation detail is described in the supplemental material.

**Figure 7:**
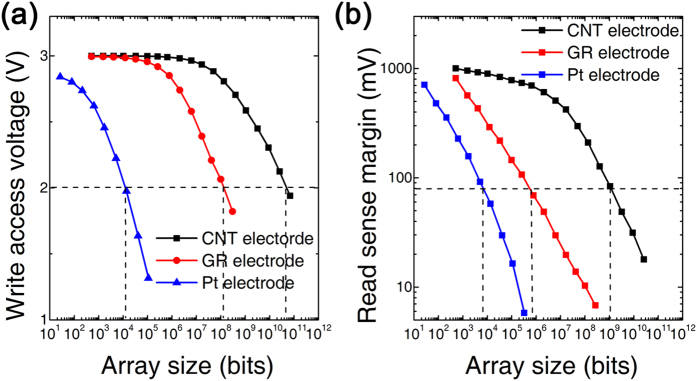
3D RRAM array performance with Pt, graphene (GR) and CNT edge electrode. (**a**) The write access voltage of selected cell. (**b**) The read sense margin with a criterion of 80 mV.

Figure 7 show the performance of 3D RRAM with three types of edge electrodes at array level. The write access voltage versus array size in Fig. 7(a) shows the advantage of device with graphene and CNT edge electrodes. An obvious decline could be observed at 10^4^ bits for device with Pt edge electrode. In contrast, for graphene and CNT edge electrode devices, the voltage degrade to 2 V when the array sizes increase to 10^8^ bits and 10^10^ bits. The decrease of write access voltage mainly comes from the interconnect resistance. With the array size increase, the equivalent resistance of interconnect is comparable with RRAM cells, which causes the divided voltage on selected cell decrease. Therefore the 3D RRAM with Pt edge electrode has the limited array size due to small HRS/LRS resistances. For 3D RRAM cells with graphene and CNT edge electrodes, the HRS/LRS resistances are 1000X larger. This property offers much large array size without write access voltage degradation.

Figure 7(b) shows the read sensing margin versus array size. By measuring the voltage difference in a 3D RRAM array when the selected cell is in HRS or LRS, the read sense margin could be evaluated. For three types of devices, hundreds mV read sense margin could be achieved when the array size is small. With array size increasing, it shows a sharp read sense margin decline in Pt edge electrode device due to cross talk issue. Again, benefited from the higher resistance and nonlinearity in HRS/LRS, the performance of graphene edge electrode device is improved. However, if the minimum read sense margin V_m_ = 80 mV is set as the criterion^29^, the graphene edge electrode device could only support 10^6^ bits which is still not good enough for high density applications. With semiconducting CNT edge electrode case, owing to the built-in selector benefit, the sneak path currents could be reduced efficiently and less voltage drops on other unselected cells. As a result, more than 10^8^ bits array size could be achieved.

The simulation results show graphene/CNT edge electrode based 3D RRAM have better performance at array level, comparing with metal edge electrode based 3D RRAM. In addition, some non-ideal effects, such as non-uniformity, device imperfection, RTN noise, etc. are not included in the simulation. Although these effects will not affect the comparison results, they still need to be investigated in the future. The excellent vertical and horizontal scaling limits give 3D RRAM technology great potential in high density memory applications.

## Conclusions

In this work, 3D RRAMs with graphene or CNT edge electrodes are demonstrated, to explore the vertical and horizontal scalability. In vertical direction, two-layer 3D Ta_2_O_5-x_/TaO_y_ RRAM cells with monolayer graphene as edge electrode are fabricated. 3D RRAM cells could switch normally with sub-nanometer electrode thickness. In horizontal direction, selector-layer free is realized by using 3D Ta_2_O_5-x_/TaO_y_ RRAM with semiconducting CNT edge electrode. In such case, the Schottky barriers formed at CNT/Sc and CNT/TaO_y_ contacts are served as the built-in selector. Based on the experimental and simulation results, different edge electrode material options are evaluated for high density application potential.

## Methods

### Device Fabrication

1. 3D Ta_2_O_5-x_/TaO_y_ RRAM with graphene edge electrode: 1) Synthesize and transfer the first layer graphene to SiO_2_ substrate. 2) Deposit 100 nm SiO_2_ insulating layer by RF sputtering. 3) Synthesize and transfer the second layer graphene to SiO_2_ substrate. 4) Deposit second SiO_2_ insulating layer. 5) Pattern by lithography and ICP dry etch with C_4_F_8_/Ar to form the drilled holes. 6) Deposit the Ta_2_O_5-x_/TaO_y_ TMO layer by RF reactive sputtering. 7) Pattern by lithography and deposit 30 nm Pt as the pillar electrode. 8) Pattern by lithography and deposit the 40 nm Pd as the contact metal on graphene.
2. 3D Ta_2_O_5-x_/TaO_y_ RRAM with CNT edge electrode: 1) Synthesize CNT on SiO_2_ substrate using CVD method. 2) Deposit 100 nm SiO_2_ insulating layer by sputtering. 3) Pattern by lithography and wet etch the drilled hole without damaging the CNT. 4) Etch the CNT using oxygen plasma in the drilled hole. 5) Deposit the Ta_2_O_5-x_/TaO_y_ TMO layer by reactive sputtering. 6) Pattern by lithography and deposit 30 nm Pt as the pillar electrode. 7) Pattern by lithography and deposit the 40 nm Sc as the contact metal on CNT.

### Material Synthesis

1. Synthesize and transfer the graphene edge electrode. Pt foils were heated to 1050 °C in H_2_ ambience. Then the reaction gases composed by CH_4_ and H_2_ were introduced into the growth quartz chamber with a total gas flow rate of 800sccm. The ratio of CH_4_:H_2_ is 0.0078:1 and the substrates were soaked in the reaction gas mixture for 60 minutes before cooling down step. The synthesized graphene films were transferred to SiO_2_ substrates by an electrochemical method^23^.
2. Synthesize the CNT edge electrode: The CNTs were synthesized by a CVD method^26^. Using ethanol and water as feed gases and Fe-Mo catalysts which were suspended in the air, the ultra-long CNT were grown directly on the SiO_2_ substrates. After the growth of CNTs, the samples were annealed at 80 °C in a low pressure environment for 5 min to make the substrates more hydrophobic.

### Characterization Techniques

The quality of graphene was characterized by Raman spectra (RENISHAW RM2000). The CNTs were observed using TEM (FEI Tecnai TF20) and SEM (QUANTA FEG450). The 3D RRAM cross-section structures were investigated by TEM, EELS (FEI Tecnai G2 F20) and FIB (FEI Nova600). The electrical characteristics were tested by Agilent B1500A semiconductor parameter analyzer and Agilent 81110A pulse generator with Cascade Summit 11000 probe station.

## Additional Information

**How to cite this article**: Bai, Y. *et al.* Stacked 3D RRAM Array with Graphene/CNT as Edge Electrodes. *Sci. Rep.* **5**, 13785; doi: 10.1038/srep13785 (2015).

## Supplementary Material

Supplementary Information
