# Supplementary material for: Stacked 3D RRAM Array with Graphene/CNT as Edge Electrodes
Source: Sci Rep. 2015 Sep 8;5:13785. doi: 10.1038/srep13785 (PMC4562297; doi:10.1038/srep13785)
Supplement: Supplementary Information [file srep13785-s1.doc]

Stacked 3D RRAM Array with Graphene/CNT as Edge Electrodes

Yue Bai, Huaqiang Wu,*, Kun Wang, Riga Wu, Lin Song, Tianyi Li, Jiangtao Wang, Zhiping Yu, He Qian

*wuhq@mail.tsinghua.edu.cn

SUPPLEMENTARY INFORMATION

1. The architecture of CNT edge electrode based 3D RRAM array


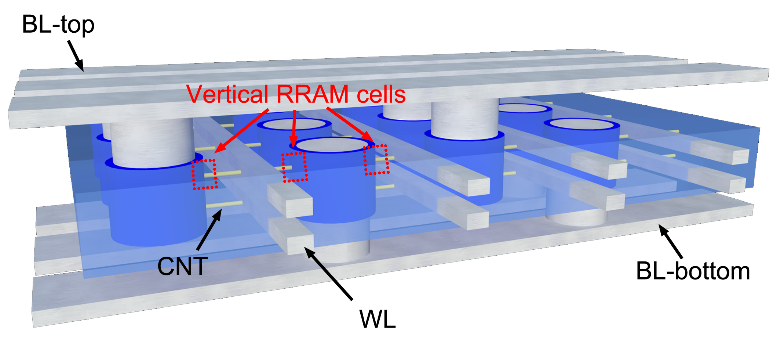


Figure S1 The schematic of CNT electrode based 3D RRAM.

The CNT edge electrode based 3D RRAM array could be achieved as shown in figure S1. Each RRAM cell could be accessed by selecting one Bit line (BL) and one word line (WL).

1. The simulation of 3D RRAM array

The basic 3D RRAM array structure is shown in Figure S2, the target cell could be chosen by selecting corresponding BL and WL.


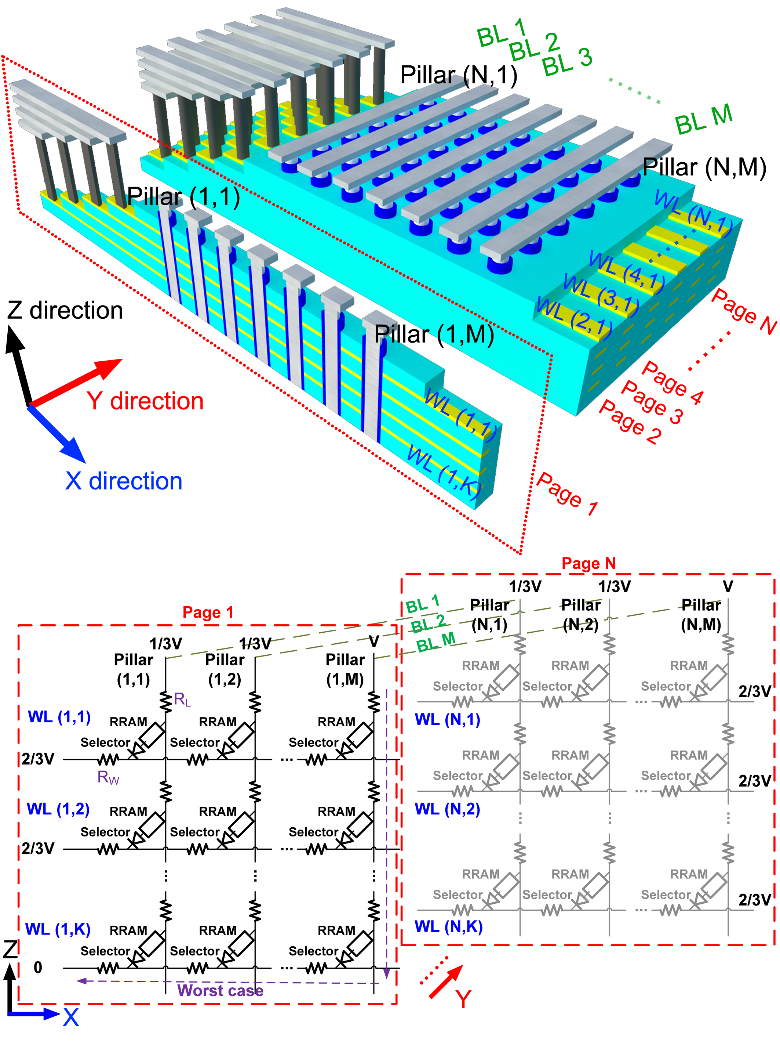


Figure S2 The physical structure and corresponding circuit diagram of 3D RRAM

The calculation of the total tolerant array size could be divided into two parts: 1) the tolerant page size and 2) the number of pages which refers to the BL length. The write access margin and read sense margin with page size and BL length is analyzed as shown in Figure S3 and Figure S4.


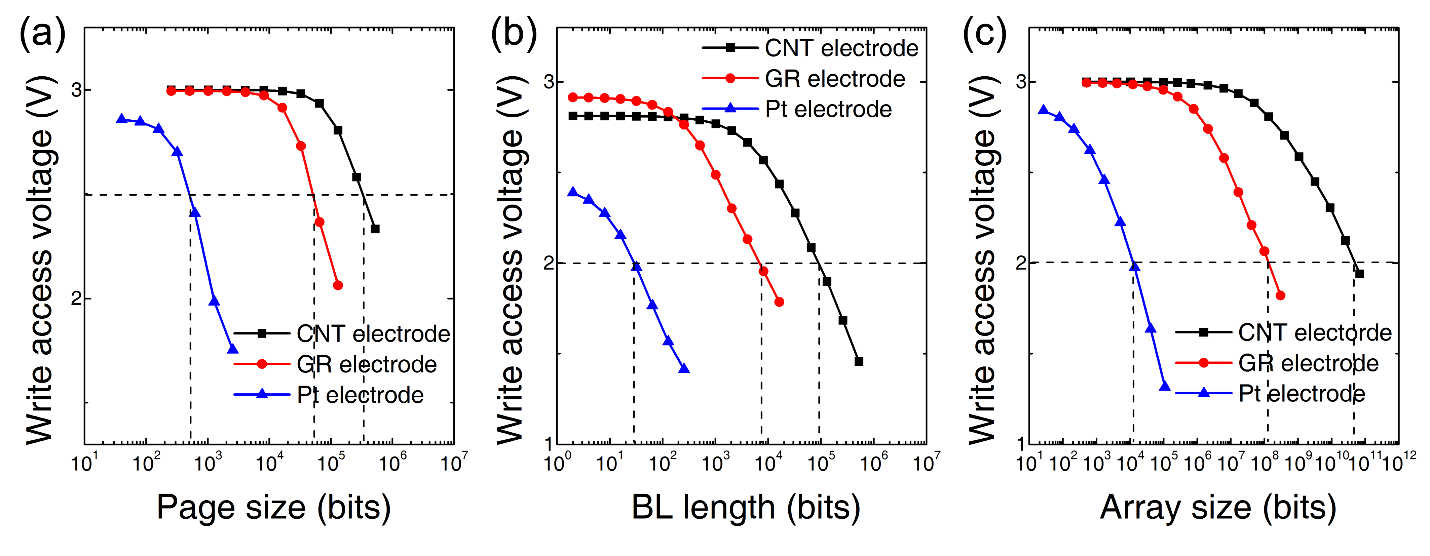


Figure S3 (a) Write access voltage versus page size. (b) Write access voltage versus BL length. (c) Write access voltage versus array size.


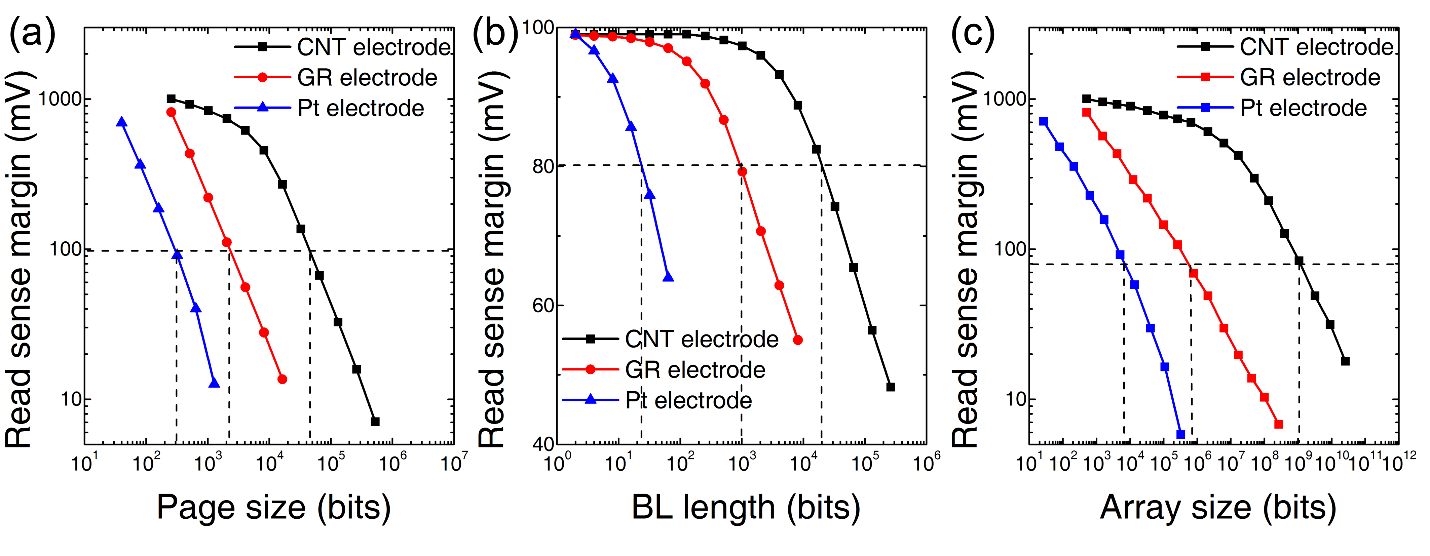


Figure S4 (a) Read sense margin versus page size. (b) Read sense margin versus BL length. (c) Read sense margin versus array size.

Table 1 SIMULATION PARAMETERS

| Design parameters | Pt BE | Graphene BE | CNT BE |
| --- | --- | --- | --- |
| WL sheet resistance | 100 Ω/□  (Metal line) | 500 Ω/□  (graphene) | 100 Ω/□  (Metal line) |
| CNT resistance per length | - | - | 10 kΩ/um |
| Pillar interconnect resistivity | 10 μΩ cm | 10 μΩ cm | 10 μΩ cm |
| BL interconnect resistivity | 10 μΩ cm | 10 μΩ cm | 10 μΩ cm |
| Contact resistance | - | 5 MΩ | 50 MΩ |
| RRAM LRS resistance | 10 kΩ | 5 MΩ | 50 MΩ |
| RRAM HRS resistance | 100 kΩ | 100 MΩ | 1 GΩ |
